# Effects of Social Anxiety on Emotional Mimicry and Contagion: Feeling Negative, but Smiling Politely

**DOI:** 10.1007/s10919-017-0266-z

**Published:** 2017-09-25

**Authors:** Corine Dijk, Agneta H. Fischer, Nexhmedin Morina, Charlotte van Eeuwijk, Gerben A. van Kleef

**Affiliations:** 1 Department of Clinical Psychology, University of Amsterdam, Postbus 15933, 1001 NK Amsterdam, The Netherlands; 2 Department of Social Psychology, University of Amsterdam, Postbus 15933, 1001 NK Amsterdam, The Netherlands; 3 Department of Psychology, University of Münster, Münster, Germany; 4 Grip, Psychology Practice, Amsterdam, The Netherlands

**Keywords:** Social anxiety, Emotional mimicry, Emotional contagion, Facial expression

## Abstract

Socially anxiety may be related to a different pattern of facial mimicry and contagion of others’ emotions. We report two studies in which participants with different levels of social anxiety reacted to others’ emotional displays, either shown on a computer screen (Study 1) or in an actual social interaction (Study 2). Study 1 examined facial mimicry and emotional contagion in response to displays of happiness, anger, fear, and contempt. Participants mimicked negative and positive emotions to some extent, but we found no relation between mimicry and the social anxiety level of the participants. Furthermore, socially anxious individuals were more prone to experience negative emotions and felt more irritated in response to negative emotion displays. In Study 2, we found that social anxiety was related to enhanced mimicry of smiling, but this was only the case for polite smiles and not for enjoyment smiles. These results suggest that socially anxious individuals tend to catch negative emotions from others, but suppress their expression by mimicking positive displays. This may be explained by the tendency of socially anxious individuals to avoid conflict or rejection.

## Introduction

Paying attention to the emotions of others generally helps people to socially coordinate and smoothen their interactions (Fischer and Manstead 2016; Keltner and Haidt 1999; Parkinson 1996, 2011; Van Kleef 2009). One observation is that individuals tend to experience similar emotions as the ones they observe, a phenomenon referred to as emotional contagion (Hatfield et al. 1993). Emotional contagion can occur through the automatic mimicry of facial displays of others’ emotional expressions (Hatfield et al. 1993). Both mimicry and contagion are seen as indicative of the tendency to empathize with another person. Indeed, mimicking others’ emotions signals affiliation and increases sympathy and rapport (e.g., Hess and Fischer 2013; McIntosh 2006). Studies on social anxiety have suggested that socially anxious individuals have more emotional problems than healthy controls, both in terms of regulating their own negative emotions and in developing positive interpersonal connections (e.g., Alden and Taylor 2004; Brown et al. 1998). Here we investigate one potential explanation for these problems, namely a deviation in emotional mimicry (i.e., to show similar facial expressions) and a deviation in emotional contagion (i.e., the tendency to experience similar emotions as others).

There are different lines of research that suggest a connection between social anxiety and emotional mimicry and contagion. First, although socially anxious individuals are able to decode basic emotions in a similar way as individuals without social anxiety (e.g., Philippot and Douilliez 2005), previous research has suggested that they still have a lower threshold for decoding (negative) emotions (Button et al. 2013; Joormann and Gotlib 2006). This may lead to poorer understanding of others’ emotions and thus decrease emotional mimicry and contagion. Second, anxious individuals commonly report experiencing less positive as well as more negative emotions compared to healthy individuals (Brown et al. 1998; Kashdan and Breen 2008; Kashdan and Steger 2006; Kashdan 2007; Watson et al. 1988; Werner et al. 2011), and studies have shown that individuals who experience negative emotions are more likely to have deficiencies in their mimicry (Likowski et al. 2011; Moody et al. 2007). Third, socially anxious individuals may behave in ways that reduce the amount of social contact with others (Leary and Kowalski 1997). Combined with evidence that individuals who do not wish to affiliate mimic less (Johnston 2002; Lakin et al. 2008; Yabar et al. 2006), this reduced tendency to affiliate may also show in a reduced tendency to emotionally mimic. Finally, with respect to emotional contagion, studies examining the neural correlates of emotional processing hint that social anxiety might be related to enhanced negative feelings while observing negative emotions of others (cf. Etkin and Wager 2007). This implies that socially anxious participants would show enhanced negative, but not enhanced positive contagion. Thus, compared to healthy controls, socially anxious individuals may be differently affected by others’ emotions, and may therefore not benefit from the positive affiliative effects of mimicry and emotional contagion (cf. Keltner and Kring 1998; Van Kleef 2016). In line with this suggestion, some studies showed that interactions with socially anxious individuals are perceived as less smooth and enjoyable (e.g., Alden and Wallace 1995; Pilkonis 1977; Voncken et al. 2008; Voncken and Dijk 2013). In the current paper, we examine whether social anxiety is related to a deficiency in the mimicry and contagion of others’ emotions.

Only a few studies to date have examined facial responses of socially anxious individuals, and the results from these studies are inconclusive. In one line of research, individuals with and without public speaking anxiety (a specific subtype of social anxiety) watched pictures of faces displaying anger and happiness, and their responses to the pictures were measured using facial electromyographic (fEMG). Two such studies showed that anxious participants tended to respond with enhanced negative expressions (i.e., *Corrugator* activity) towards others’ negative emotional displays and with diminished positive expressions (i.e., *Zygomatic Major* activity) towards others’ positive emotional displays (Dimberg 1997; Vrana and Gross 2004). Another study replicated these findings for negative expressions, but not for positive expressions. On the contrary, the results of this study showed that socially anxious individuals also displayed increased positive facial reactions to positive expressions (Dimberg and Thunberg 2007). In yet another study, socially anxious individuals only showed less positive expression in reaction to positive faces, but no enhanced negative expressions to negative faces (Dimberg and Christmanson 1991). Finally, using observer ratings instead of fEMG, Heerey and Kring (2007) examined smiling and frowning of participants with different degrees of social anxiety during actual interaction. Their results showed that highly socially anxious participants hardly mimicked enjoyment smiles (including facial actions of both the mouth and the eyes), whereas there were no differences between high and low anxiety participants’ displays of polite smiles (involving the mouth only) and frowns.

It is evident from these mixed findings that the role of social anxiety in shaping facial mimicry is poorly understood. Also, the role of social anxiety in emotional contagion has hardly been examined. In addition, inconsistent results from previous studies may be due to differences in methodology, such as participant selection or the nature of the stimuli. Against this background, the goal of the present studies was to examine the effects of social anxiety on mimicry as well as contagion for different emotions, using different experimental procedures.

The current studies differ in two important ways from previous studies on social anxiety and facial mimicry. One difference is that we used computerized facial coding (using the Computer Expression Recognition Toolbox; Littlewort et al. 2011) to measure participants’ facial expressions, instead of fEMG. We used this automatic coding software because we are interested in studying facial mimicry during an actual social interaction (in Study 2), and we expected that hooking participants up to facial electrodes might attenuate the results by hampering the naturalness and smoothness of the interaction. Furthermore, we only aimed to measure muscle movements that are clearly visible to the eye (cf. Sato and Yoshikawa 2007), and therefore the measurement of very subtle movements that can only be detected by fEMG was not necessary. Another difference from previous studies is that we used dynamic expressions instead of still photographs, because dynamic expressions might also better reflect what actually occurs in interactions.

The inconsistent results in the extant literature suggest two alternative predictions regarding the effects of social anxiety on mimicry. One possibility is that the greater proclivity of socially anxious individuals to experience negative emotional states (Brown et al. 1998; Watson et al. 1988) reduces their tendency to mimic and catch others’ emotions, given evidence that negative moods can hamper facial mimicry (Likowski et al. 2011; Moody et al. 2007). This implies that socially anxious participants would show reduced emotional mimicry for all emotions. An alternative possibility, however, is that, irrespective of what they are actually feeling, socially anxious people do not always show reduced mimicry, but mimic others’ positive expressions more (Dimberg and Thunberg 2007), in order to enhance their acceptance by others (Heerdink et al. 2015).

## Study 1

In Study 1, we examined participants’ emotional and facial reactions towards short movie clips of people displaying a neutral face, happiness, contempt, fear, and anger. The vast majority of studies that tested fEMG of participants who were high and low on public speaking anxiety have only focused on anger versus happiness (Dimberg 1997; Dimberg and Christmanson 1991; Dimberg and Thunberg 2007; Vrana and Gross 2004). By including two additional negative emotions, we can examine whether any differential effects of social anxiety on mimicry and/or emotional contagion occur along the lines of positive versus negative valence or follow a more discrete emotion pattern.

The study has a non-directional hypothesis concerning the influence of social anxiety on the facial reactions of the participants: we hypothesize that socially anxious individuals show atypical mimicry in response to the facial displays. In addition, we explore whether this is different for different discrete emotions. Second, we hypothesize socially anxious individuals also show an atypical pattern of emotional contagion, reflected enhanced negative, but not enhanced positive contagion.

### Method

#### Participants

Based on previous studies, a medium to large effect of mimicry and contagion could be expected (e.g., Dimberg 1997; Khvatskaya and Lenzenweger 2016; Vrana and Gross 2004). For a 2 (group) by 5 (emotion) interaction effect, with an expected effect size of f = .25, a power of .95, and an expected correlation of .5 between the repeated measures, a total sample size of *N* = 32 would have been sufficient. In Study 1, participants were 105 first-year psychology students who received course credits or a compensation of 7€. Their mean age was 21 (*SD* = 6) and 82% were female. Participants were invited to participate in the study based on their score on the Social Interaction Anxiety Scale (SIAS; Mattick and Clarke 1998; see description below), which all first-year psychology students have to complete during a mass screening at the beginning of their program. We specifically invited individuals who were in the highest 25% and the lowest 50% range of SIAS scores to ensure that there would be sufficient variability in our sample. We invited the 50% lowest SIAS scores, instead of just the 25% lowest, because extremely fearless individuals may be dysfunctional as well (cf. Hofmann and DiBartolo 2010). To validate our selection, participants filled out the SIAS a second time at the start of the current study. The test–retest correlation of the SIAS was good, *r*(100) = .82, *p* < .001 (three participants did not consent to a link between the current data and their scores obtained during the mass screening and were therefore not included in this particular analysis). Nevertheless, the test–retest analyses showed that a considerable number of the participants with more extreme scores during the first measurement scored more on average during the second measurement (i.e., regression towards the mean). The distribution of this second measurement resembled a continuous scale more than the original dichotomous variable. Still, the participants’ SIAS scores showed considerable variation (*M* = 17.93, *SD* = 13.77, range 0–65) and 14.3% of participants had a score higher than 35, which can be considered in the phobic range (Heinrichs et al. 2002). Therefore, the second measure of the SIAS was used as a continuous measure of social anxiety in the current study. Eleven of the 105 participants’ recordings showed technical errors (e.g., darkness, angle) that made coding impossible. Therefore, the analyses of facial expressions were conducted with the remaining 94 participants.

#### Procedure

Before the study started, participants gave informed consent. Then participants were asked to complete several questionnaires.^1^ Next, participants were instructed to watch short videos of actors displaying no emotion (neutral), anger, fear, happiness, and contempt (the *stimulus emotions)*, which were selected from the Amsterdam Dynamic Facial Expression Set (van der Schalk et al. 2011b). To enhance attention and involvement, participants were instructed to try to read the thoughts of the actors in the videos and were informed that they would have a conversation with one of the actors following the experiment. Similar to previous studies testing mimicry, the participants saw six models portraying the same emotion in one block (cf. Dimberg 1997; Dimberg and Christmanson 1991; Dimberg and Thunberg 2007; van der Schalk et al. 2011a; Vrana and Gross 2004). A block consisted of three male and three female actors displaying the emotion in a clip of 5 s, each intermitted by a fixation cross of one second. Thus, participants saw five emotion blocks (neutral, happy, contempt fear, and anger) of six faces, adding up to 30 film clips in total. To counterbalance order effects, we used four different versions of the task. In each version, the first block was always neutral, but the other emotion blocks were presented in different orders. Each emotion video was approximately 5 s long, starting with a neutral expression and reaching the apex after approximately 2 s. The participants were filmed while watching the short videos, so that their facial expressions could be coded afterward. After each emotion block, participants were asked about their current emotional state. At the end of the study, we debriefed the participants and told them that they would not have a conversation with one of the actors.

### Measures

#### Questionnaires

To measure social anxiety, participants completed the Social Interaction Anxiety Scale (Mattick and Clarke 1998). The SIAS is a 20-item, self-report scale that measures fear of social interaction situations. Good internal consistency, test–retest reliability, and validity have been reported for the SIAS (Orsillo 2001).

#### Facial Expressions

While watching the videos, participants’ faces were recorded with a small camera that was placed on a tripod, so that it was just above the computer screen. To code facial expressions, we used the Computer Expression Recognition Toolbox (CERT; Littlewort et al. 2011). This program is based on Ekman and Friesen’s (1978) Facial Action Coding System (FACS). CERT gives an Action Unit (AU) score for each video frame. Output from CERT can be interpreted as estimates of the AU intensities and are significantly correlated with the intensity of facial actions, as coded by FACS experts (Bartlett et al. 2006; Littlewort et al. 2011). The program has been used in previous studies examining facial reactions towards social stimuli (e.g., Khvatskaya and Lenzenweger 2016; Schaafsma et al. 2015). For each emotion, the most obvious expressions of the upper and the lower face that were displayed in the videos of the stimulus emotions were selected. For happiness, these were the Cheek Raiser (AU6) and the Lip Corner Puller (AU12), for contempt the Outer Brow Raiser (AU2) and the Dimpler (AU14), for fear the Upper Lid Raiser (AU5) and Lip Stretcher (AU20), and for anger the Brow Lowerer (AU4) and the Lip Tightener (AU23). For each emotion block, a mean AU score was calculated, averaged across all frames during the block (35 s * 29 frames = 1015 frames).

#### Experienced Emotions

After each emotion block, participants were asked to indicate on five visual analogue scales (VAS) to what extent they felt happy, contemptuous, nervous, and irritated. Participants were asked about irritation and nervousness instead of anger and fear because these latter labels might reflect too much intensity for the current situation, which could reduce variation on these scales (cf. Izard et al. 1974). The scales ranged from 0 (*not at all*) to 100 (*very much*).

### Results

#### Mimicry

Participants’ use of the two facial movements associated with the stimulus emotion were analyzed using mixed-model MANOVA, with the within factor stimulus emotion (relevant emotion vs. one of the other emotions) as a repeated measure and social anxiety (measured with the SIAS) as a continuous predictor (the SIAS was centered for this purpose). In case of a significant main effect of stimulus emotion, we examined these further by comparing the relevant emotion with each of the other four emotions, adjusting the significance level with a Bonferroni correction (thus α = .05/4 = .0125). For example, the actors in the movie clips of happiness clearly expressed AU6 (Cheek Raiser) in the upper face and AU12 (Lip Corner Puller) in the lower face. Thus, the participants’ displays of AU6 and AU12 while watching happiness were compared with the displays of AU6 and AU12 while watching any of the other emotions in the video (neutral, contempt, fear, or anger). The means and standard deviations of the expressions are presented in Table 1. To provide insight in the direction of a possible effect of social anxiety, Table 1 also reports the correlations of the expressions with the SIAS score.

**Table 1:** Means (and standard deviations) of the coded expression and experienced emotions and their correlation with the SIAS (study 1)

	Expression displayed in clip									
	Neutral		Happy		Contempt		Fear		Anger	
	M (SD)	r_sias_	M (SD)	r_sias_	M (SD)	r_sias_	M (SD)	r_sias_	M (SD)	r_sias_
Happy										
Cheek raiser (AU6)	.20 (.24)	.04	.29 (.23)	.12	.26 (.26)	.14	.27 (.27)	.12	.26 (.27)	.05
Lip corner puller (AU12)	− .44 (.70)	− .14	− .04 (.69)	− .08	− .20 (.65)	− .11	− .17 (.72)	− .09	− .30 (.66)	− .20
Feeling happy	45.99 (17.59)	− .20**	75.45 (15.22)	− .08	50.74 (21.27)	− .20*	48.11 (21.76)	− .21**	45.79 (24.99)	− .25**
Contempt										
Outer brow Raiser (AU2)	− .25 (.30)	.08	− .24 (.31)	.12	− .21 (.31)	.12	− .23 (.32)	.17	− .24 (.27)	.18
Dimpler (AU14)	1.09 (.48)	− .16	1.23 (.55)	− .13	1.14 (.51)	− .01	1.13 (.47)	− .12	1.10 (.48)	− .11
Feeling contempt	11.66 (48.53)	.09	6.44 (11.47)	.16	21.59 (22.17)	.17	12.87 (16.10)	.12	14.51 (18.49)	.16
Fear										
Upper lid raiser (AU5)	− .54 (.15)	.08	− .55 (.13)	.06	− .56 (.13)	.09	− .55 (.14)	.13	− .55 (.13)	.07
Lip stretcher (AU20)	1.34 (.41)	− .06	1.33 (.39)	− .02	1.33 (.49)	− .06	1.34 (.45)	− .04	1.36 (.44)	− .14
Feeling nervous	22.65 (21.85)	.39**	9.26 (13.03)	.50**	17.80 (21.10)	.46**	21.84 (22.46)	.49**	19.34 (22.26)	.34**
Anger										
Brow lowerer (AU4)	− .01 (.17)	.18	− .05 (.19)	.12	− .02 (.20)	.12	− .03 (.19)	.10	− .03 (.19)	.13
Lip tightener (AU23)	1.01 (.33)	− .19	.97 (.34)	− .14	.99 (.36)	− .14	1.01 (.31)	− .13	.98 (.36)	− .18
Feeling irritation	12.10 (14.91)	.16	5.86 (10.03)	.16	22.57 (23.56)	.27**	15.08 (18.49)	.25**	21.24 (23.88)	.29**

For the correlations with the coded expressions (AUs) the *df* = 92, for the correlations with the experienced emotions the *df* = 103

* *p* < .05

** *p* < .01

For *happiness*, the analysis showed a main effect of stimulus emotion, *F*(8, 85) = 7.12, *p* < .001, η_p_^2^ = .40. There was no significant effect of social anxiety, *F*(2, 91) = 2.87, *p* = .061, η_p_^2^ = .06, and no interaction between stimulus emotion and social anxiety, *F*(8, 85) = .40, *p* = .917, η_p_^2^ = .04. Follow-up analysis showed that the effect of stimulus emotion was significant for both AU6, *F*(4, 368) = 7.15, *p* < .001, η_p_^2^ = .07, and AU12, *F*(4, 368) = 11.21, *p* < .001, η_p_^2^ = .11. The follow-up analyses further showed that participants displayed more AU12 while watching happiness than neutral, *F*(1, 92) = 42.61, *p* < .001, η_p_^2^ = .32, contempt, *F*(1, 92) = 8.73, *p* = .004, η_p_^2^ = .09, or anger, *F*(1, 92) = 14.47, *p* < .001, η_p_^2^ = .14, but not fear, *F*(1, 92) = 5.08, *p* = .027, η_p_^2^ = .05 (see Table 1). For AU6, only the contrast with neutral was significant, *F*(1, 92) = 40.70, *p* < .001, η_p_^2^ = .31. The contrasts with contempt, *F*(1, 92) = 2.03, *p* = .158, η_p_^2^ = .02, fear, *F*(1, 92) = 1.33, *p* = .252, η_p_^2^ = .01, and anger, *F*(1, 92) = 2.60, *p* = .110, η_p_^2^ = .03 were not significant.

The analyses of *contempt* showed no main effect of stimulus emotion, *F*(8, 85) = 1.54, *p* = .156, η_p_^2^ = .13, no effect of social anxiety, *F*(2, 91) = 2.14, *p* = .124, η_p_^2^ = .05, and no interaction, *F*(8, 85) = .70, *p* = .687, η_p_^2^ = .06.

Likewise, the analyses of *fear* showed no main effect of stimulus emotion, *F*(8, 85) = .95, *p* = .484, η_p_^2^ = .08, no effect of social anxiety, *F*(2, 91) = .95, *p* = .390, η_p_^2^ = .02, and no interaction, *F*(8, 85) = .45, *p* = .891, η_p_^2^ = .04.

For *anger*, the analysis showed a main effect of stimulus emotion, *F*(8, 85) = 2.84, *p* = .008, η_p_^2^ = .21. However, there was no effect of social anxiety, *F*(2, 91) = 2.19, *p* = .118, η_p_^2^ = .05, and no interaction, *F*(8, 85) = .25, *p* = .979, η_p_^2^ = .02. A follow-up analysis to examine the effect of stimulus emotion showed that this effect was significant for both AU4, *F*(4, 368) = 2.52, *p* = .041, η_p_^2^ = .03, and AU23, *F*(4, 368) = 3.17, *p* = .014, η_p_^2^ = .03. However, the follow-up analysis of AU23 showed that this was driven by a non-significant effect that was in the opposite direction than expected: Participants tightened their lips more while watching neutral expressions than while watching anger expressions, *F*(1, 92) = 6.48, *p* = .013, η_p_^2^ = .07 (see Table 1). The display of AU23 did not differ between watching angry and happy, *F*(1, 92) = .08, *p* = .774, η_p_^2^ < .01, contempt, *F*(1, 92) = .06, *p* = .814, η_p_^2^ < .01, or fearful *F*(1, 92) = 1.84, *p* = .178, η_p_^2^ = .02 expressions. For AU4, planned contrasts showed that participants showed more AU4 while watching anger than while watching happiness *F*(1, 92) = 12.93, *p* = .001, η_p_^2^ = .12 (see Table 1). The other contrasts with neutral, *F*(1, 92) = .31, *p* = .581, η_p_^2^ < .01, contempt, *F*(1, 92) = 1.25, *p* = .267, η_p_^2^ = .01, and fear, *F*(1, 92) = 4.32, *p* = .040, η_p_^2^ = .05, were not significant.

#### Self-Reported Emotions

The emotions that participants experienced while watching the clips were analyzed per experienced emotion using mixed-model ANOVA, with the stimulus emotion as a within-subjects factor (relevant emotion vs. one of the other emotions) and the centered SIAS score as a continuous predictor. In case of significant effects of stimulus emotion, we examined these further by comparing the relevant stimulus emotion to the other four emotions (again using α = .05/4 = .0125 as a significance level). For example, the amount of happiness felt after watching a happy clip was compared to the amount of happiness felt after watching neutral, anger, contempt, or fear. The means and standard deviations of the felt emotions, as well as the correlations with the SIAS, are presented in Table 1.

For *happiness* there was a main effect of stimulus emotion, *F*(4, 412) = 70.16, *p* < .001, η_p_^2^ = .41, and also a main effect of social anxiety, *F*(1, 103) = 6.56, *p* = .012, η_p_^2^ = .41. There was no interaction between stimulus emotion and social anxiety, *F*(4, 412) = 1.57, *p* = .182, η_p_^2^ = .02. The correlations of happiness with the SIAS showed that social anxiety was negatively correlated with happiness. The follow-up contrasts showed that participants felt significantly happier while watching happiness than while watching any of the other emotions (vs. neutral, *F*(1, 103) = 173.25, *p* < .001, η_p_^2^ = .63, contempt, *F*(1, 103) = 117.59, *p* < .001, η_p_^2^ = .53, fear, *F*(1, 103) = 134.94, *p* < .001, η_p_^2^ = .57, and anger, *F*(1, 103) = 123.19, *p* < .001, η_p_^2^ = .55; see Table 1).

For *contempt*, only the effect of stimulus emotion was significant, *F*(4, 412) = 25.14, *p* < .001, η_p_^2^ = .20. There was no significant effect of social anxiety, *F*(1, 103) = 2,99, *p* = .087, η_p_^2^ = .03, and no interaction effect, *F*(4, 412) = .80, *p* = .524, η_p_^2^ = .01. The contrast for stimulus emotion showed that participants felt more contempt while watching contempt than any of the other emotions (vs. neutral, *F*(1, 103) = 26.64, *p* < .001, η_p_^2^ = .21, happiness, *F*(1, 103) = 60.22, *p* < .001, η_p_^2^ = .37, anger, *F*(1, 103) = 28.88, *p* < .001, η_p_^2^ = .22 and contempt, *F*(1, 103) = 15.79, *p* < .001, η_p_^2^ = .13).

For *nervousness* there was a main effect of stimulus emotion, *F*(4, 412) = 15.88, *p* < .001, η_p_^2^ = .14. There also was a main effect of social anxiety, *F*(1, 103) = 39.84, *p* < .001, η_p_^2^ = .28, but there was no interaction between stimulus emotion and social anxiety, *F*(4, 412) = 1.67, *p* = .157, η_p_^2^ = .02. The correlations with the SIAS showed that social anxiety was positively correlated with nervousness. The contrasts showed that participants felt more nervous while watching fear than while watching happiness, *F*(1, 103) = 40.09, *p* < .001, η_p_^2^ = .28. There was no difference between the amount of nervousness while watching fear versus watching neutral, *F*(1, 103) = .18, *p* = .675, η_p_^2^ < .01, contempt, *F*(1, 103) = 6.39, *p* = .013, η_p_^2^ = .06, or anger, *F*(1, 103) = 1.73, *p* = .192, η_p_^2^ = .02.

For *irritation* there was a main effect of stimulus emotion, *F*(4, 412) = 25.54, *p* < .001, η_p_^2^ = .20. The contrast analyses showed that participants felt more irritation while watching anger than neutral, *F*(1, 103) = 18.06, *p* < .001, η_p_^2^ = .15, happiness, *F*(1, 103) = 54.36, *p* < .001, η_p_^2^ = .35, and fear *F*(1, 103) = 7.73, *p* = .006, η_p_^2^ = .07, but not contempt, *F*(1, 103) = .33, *p* = .566, η_p_^2^ < .01. There also was a main effect of social anxiety, *F*(1, 103) = 10.72, *p* = .001, η_p_^2^ = .10, and an interaction between stimulus emotion and social anxiety, *F*(4, 103) = 2.89, *p* = .022, η_p_^2^ = .30. The correlations with social anxiety showed that the more socially anxious participants were, the more irritation they reported feeling in reaction to all three negative emotions. The correlation between social anxiety and felt irritation was not significant while watching neutral or happiness (see Table 1).

### Discussion

With respect to emotional contagion, we found—in line with previous studies—that participants reported more happiness, nervousness, contempt, and irritation in response to the same emotion displays. We also found that socially anxious individuals reported to experience more irritation in response to all negative emotions (fear, anger, contempt). Furthermore, more generally, social anxiety was related to experiencing less happiness and more nervousness, irrespective of what was observed in others. We would like to point out, that more socially anxious participants appeared just as able to “catch” happiness from others; they were just generally less happy. This latter finding, therefore, seems to reflect a general response rather than a signal of emotional contagion, which is a more specific response to another’s emotional display.

We did not find support for our hypothesis that social anxiety would affect mimicry patterns, as we did not find any interaction between social anxiety and facial responses to the specific emotion displays. The general results for emotional mimicry were in line with previous studies, however. Participants frowned more while observing angry versus happy faces, and smiled more while observing happy faces, compared to faces showing negative emotions (e.g., Dimberg 1997; Vrana and Gross 2004). Our results thus did not show differential mimicry reactions for the other emotions. Specifically, we did not find any mimicry of fear and contempt. Whereas some previous studies showed activation of the muscles in the forehead (*Lateral Frontalis*) in response to observing fear (Moody et al. 2007; Rymarczyk et al. 2016), we did not find mimicry of the widening of the eyes or a lip stretch, which were the main expressions in our stimuli. An explanation for the lack of mimicry in these emotions, is that they do not signal affiliation (Hess and Fischer 2013), which is an important requirement for mimicry. A second explanation is more technical, namely that certain facial movements are less likely to be detected with CERT than others. A frown is clearly visible, and thus detectable, whereas the Outer Brow Raiser (AU2), the Upper Lid Raiser (AU5) and Lip Stretcher (AU20) may have required more pronounced movements in order to be detected. An explanation for the finding that all negative emotions elicited the same amount of frowning as anger, is that this expression is a reaction to the negative valence of the emotional stimulus rather than mimicry of the discrete emotion of anger (see also Hess and Fischer 2013). Since neutral was always the first expression the participants saw, the frown related to observing these clips may also have reflected concentration or confusion about the task (Rozin and Cohen 2003).

The finding that socially anxious individuals do not respond with atypical mimicry is in contrast with the findings of previous studies using fEMG to study mimicry in social anxiety, which showed mixed findings with regard to the direction of the effect, but often did find differential effects for social anxiety (e.g., Dimberg 1997; Dimberg and Christmanson 1991; Dimberg and Thunberg 2007; Heerey and Kring 2007; Vrana and Gross 2004). As mentioned above, it is possible that these fEMG studies detected very subtle muscular movements that are not outwardly visible and cannot be picked up by face-reading software. Our results suggest that visible mimicry is not deficient in socially anxious individuals: Interaction effects that would reveal a moderating role of social anxiety on facial mimicry were non-significant, and the corresponding effect sizes were very small. Thus, our findings suggest that socially anxious individuals are not different from individuals without social anxiety in their outwardly visibly mimicry of others’ emotional expressions, at least in the non-interactive setting that we studied in this experiment.

## Study 2

In Study 1 we examined facial expressions in reaction to standardized stimuli in a controlled setting. However, there are several reasons why being involved in an actual interaction may produce different results (cf. Heerey and Kring 2007). First, individuals with social anxiety usually do not experience anxiety when they are alone, watching film clips on a computer screen. They probably do feel anxious when involved in social interactions, since fear of interactions is the core of this disorder (American Psychiatric Association 2013). It is conceivable that it is the actual experience of fear that affects mimicry of facial expressions (e.g., Dimberg 1986; Moody et al. 2007). In addition, cognitive behavioral models of social anxiety disorder predict that anxiety elicits safety behaviors, implying that individuals with social anxiety refrain from using social skills, which they actually do possess, because they fear that this behavior might lead to rejection (e.g., Clark and Wells 1995). Therefore, Study 2 was designed to examine the effect of social anxiety on facial mimicry (i.e., smiles and frowns—see Task and Procedure) during an actual social interaction.

The use of an interaction design also allowed us to examine the interaction partners’ judgments of the participants. Individuals with social anxiety are known to have difficulties in the interpersonal domain (Alden and Taylor 2004). If socially anxious individuals show atypical mimicry of facial expressions, this could affect their partners’ perceptions of their likeability, indicated by a reduced desire for future contact with the individual and a less positive global judgment (e.g., Voncken and Dijk 2013).

### Method

#### Participants

Because Study 2 tested an actual social interaction, we expected a slightly larger effect than in previous non-social experiments (e.g., Vrana and Gross 2004). For a 2 (group) by 2 (time) interaction effect, with an expected effect size of f = .40, a power of .95, and an expected correlation of .5 between the repeated measures, a total sample size of *N* = 24 would have been sufficient. In Study 2, participants were 46 first-year psychology students (*M*_*age*_ = 20; *SD* = 4) who received course credits or a compensation of 7€. They had not participated in Study 1. Because the assistants (interaction partners) were female, all participants were female as well. As in Study 1, participants were invited to participate in the study on the basis of their score on the Social Interaction Anxiety Scale (SIAS; Mattick and Clarke 1998), which all first-year students completed during a mass screening at the beginning of their program. We invited individuals who were in the highest 25% and the lowest 25% range of SIAS scores to ensure that there would be sufficient variability in our sample. In contrast to Study 1, we only selected the lowest 25% range (instead of the lowest 50%), because the test–retest analyses of the participants in the first study showed that a considerable number of the participants with more extreme scores during the first measurement scored on average during the second measurement (i.e., regression towards the mean). This way, we aimed to retain enough spread and a wide range in our sample. We again measured the SIAS at the beginning of the current study and used this more recent score to tap participants’ social anxiety. The test–retest correlation of these two measures was *r*(44) = .84, *p* < .001. Participants’ SIAS scores showed considerable variation (*M* = 17.52, *SD* = 11.87, range 0–46) and the sample contained 6.5% of participants with a SIAS score higher than 35, which can be considered in the phobic range (Heinrichs et al. 2002). The SIAS score was again used as a continuous variable. For one participant, the recordings showed technical errors (e.g., darkness, angle) for some expressions. Therefore, the analyses of these expressions were conducted with the remaining 45 participants.

#### Task and Procedure

Before the study started participants gave informed consent, and next they completed the SIAS. The social task that followed consisted of listening to a “research assistant” explaining the rules of a silly parlor game, using a script that she had memorized. This assistant was actually one of three professional actresses who were unaware of the aims of the study (which allowed us to ask her to judge the participants). Just before the social task started, the assistant judged the participant (see Measures: Judgment). To make the task especially provoking for socially anxious individuals, the participants were informed that they had to play this silly game later on.

During the assistant’s explanation of the task, participants were made to believe that they had to do silly things: lip-reading with a fake mustache, humming songs with a clip on their nose, and drawing farm animals while making corresponding noises. While explaining this, the assistant kept a neutral expression, except for two specific parts in the middle and at the end of the script in which the assistant displayed six frowns or six smiles at fixed moments in the instruction. The frowns occurred while the assistant explained “the grumble mike”, a children’s microphone to which the participants supposedly had to entrust whom or what they hated. The smiles were displayed when the assistant explained “my favorite memory”, in which the assistant informed the participants that they should wear a children’s tiara and talk about their favorite child memories. Only smiles and frowns were used because these expressions were relatively easy to control by the assistant and could be displayed with a rather strong intensity. The order of parts of the script with smiles and frowns was counterbalanced across participants.

To make the task more similar to computer tasks and to prevent judgments being based on other characteristics of the participants such as clothing, visual access during the social task was limited by a screen that was placed on the table. This screen had a square hole, such that the assistant and the participant could only see each other’s face, and it was presented as part of the game. While listening to the bogus instruction, both the assistant and the participants were filmed. After the instruction, the participants were asked to report their emotions and the assistant was asked to judge the participant again (see Measures: Judgment). Finally, participants learned that they would not actually play the silly game, and they were debriefed and dismissed.

### Measures

#### SIAS

As in Study 1, participants completed the Social Interaction Anxiety Scale (Mattick and Clarke 1998) to measure social anxiety.

#### Judgments of Likeability

Just before and right after the social task, the assistant was asked to quickly and globally rate the participants on a scale from 1 (*very negative*) to 10 (*very positive*), which is similar to the Dutch grading system (Voncken and Dijk 2013). To examine a change in judgment we calculated a Δ-judgment score by subtracting the score after the social task from the score just before the task. Furthermore, for a more thorough measure of the likeability of participants, we asked the assistant to complete the Desire for Future Interaction scale (DFI; Coyne 1976) after the social task. The DFI consists of eight 5-point Likert-type scales that measure the extent to which the assistant would wish to engage in future activities with the participant (α = .96). The DFI has been shown to be a reliable instrument, also in Dutch speaking samples (e.g., Voncken et al. 2010).

#### Facial Expressions

As in the first study, participants’ facial expressions were coded using the Computer Expression Recognition Toolbox (CERT; Littlewort et al. 2011). To examine the mimicry of smiles, the participants’ displays of AU12 (Lip Corner Puller) and AU6 (Cheek Raiser; indicative of enjoyment smiles) were examined; for the mimicry of frowns AU4 (Brow Lowerer) was examined. From the recordings of the participants, we selected the specific time points in which the assistant displayed the six smiles and six frowns. Across these time points we calculated the mean score of the participants’ expressions in the 50 frames (1.7 s) before and the 50 frames after the onset of the expression in the assistant. Next, a mean AU score *before* (the expression of the assistant) and a mean AU score *after* was calculated, averaging across the six frowns and smiles.

#### Self-Reported Emotions

To examine the participants’ *state social anxiety,* we asked participants to indicate on a scale from 1 to 9 to what extent they felt secure, relaxed, nervous, and anxious. In each of these items a higher score indicated more anxiety. Cronbach’s alpha was .83, allowing us to calculate a mean score. Furthermore, since the task was supposed to be funny, participants were asked to what extent they felt joyful and cheerful (on a scale from 1 to 9; *r*(44) = .47, *p* = .001). We created a mean *cheerfulness* score, with higher scores indicating more cheerfulness.

### Results

#### Mimicry

To examine the mimicry of *smiles* and *frowns,* participants’ display of AU6 (Cheek Raiser), AU12 (Lip Corner Puller) and AU4 (Brow Lowerer) were analyzed in three separate mixed-model ANOVAs, with *time* (before vs. after the expression of the assistant) as a within subject variable and with social anxiety (the centered SIAS score) as a continuous predictor. Means and standard deviations of the expressions, as well as the correlation with the SIAS, are presented in Table 2.

**Table 2:** Means (and standard deviations) of the coded expression before and after the assistant’s display and their correlation with the SIAS (Study 2)

	Before expression				After expression			
	*M* (*SD*)	*r*_*sias*_			*M* (*SD*)	*r*_*sias*_		
		*r*	*df*	*p*		*r*	*df*	*p*
Smile								
Cheek raiser (AU6)	.56 (.34)	− .06	44	.687	.82 (.40)	.06	44	.714
Lip corner puller (AU12)	.51 (.61)	− .08	44	.609	.95 (.68)	.06	44	.696
Frown								
Brow lowerer (AU4)	− .19 (.22)	.14	43	.354	− .18 (.22)	.13	43	.392

For AU4 we found a main effect of time, *F*(1, 43) = 7.46, *p* = .035, η_p_^2^ = .10. However, there was no effect of social anxiety, *F*(1, 43) = .82, *p* = .372, η_p_^2^ = .02. Also, there was no interaction between time and social anxiety, *F*(1, 43) = .33, *p* = .570, η_p_^2^ = .01. For AU6 we also found a main effect of time, *F*(1, 44) = 94.80, *p* < .001, η_p_^2^ = .68, but no effect of social anxiety, *F*(1, 44) < .01, *p* = .987, η_p_^2^ < .01. and no interaction between time and social anxiety, *F*(1, 44) = 2.43, *p* = .126, η_p_^2^ = .05. Thus, participants mimicked both AU6 and AU4 displays by the assistant (see Table 2), but this did not depend on their social anxiety level.

**Fig. 1:**
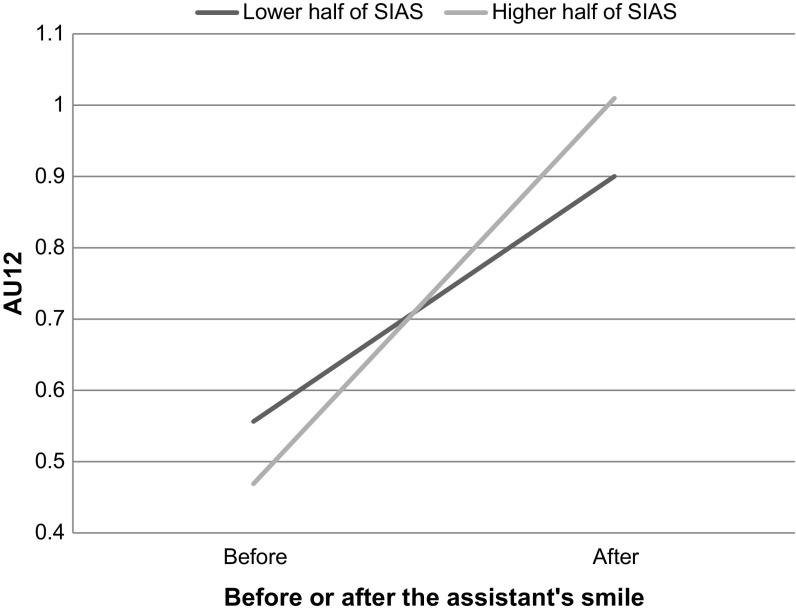
Graphical display of the interaction between social anxiety and timing for the intensity of the use of AU12 (Lip Corner Puller) using a median split of the SIAS

For AU12, we found a main effect of time, *F*(1, 44) = 106.63, *p* < .001, η_p_^2^ = .71, and no main effect of social anxiety, *F*(1, 44) = .01, *p* = .969, η_p_^2^ < .001. However, the interaction between time and social anxiety was significant, *F*(1, 44) = 4.15, *p* = .048, η_p_^2^ = .09. To examine the pattern of this interaction between a within-subjects and a continuous variable, we calculated the correlations for the two different time-points (see Table 2). Before the assistant smiled, there was as slightly negative correlation between social anxiety and AU12, but after the assistant smiled, this changed to a small positive correlation. These correlations were not significantly different from zero (see Table 2), but the interaction indicates that they differ from each other (see Fig. 1 for a graphical display of this interaction).

#### Emotions and Judgments of Likeability

The social anxiety level of the participants was not related to a reduced desire for interaction after the task, *r*_dfi_ (44) = − .16, *p* = .300, nor to a shift in the more global judgment from before to after the task *r*_Δjudgment_ (44) = − .13, *p* = .405. Furthermore, the correlation between SIAS and the state social anxiety score was *r*(44) = .56, *p* < .001, indicating that the greater participants’ dispositional social anxiety, the more socially anxious they were during the social task. The correlation between the SIAS and cheerfulness was not significant, *r*(44) = − .20, *p* = .184.

### Discussion

Study 2 was designed to make the social nature of the situation more salient, as this would make potential effects of social anxiety larger. Indeed, in contrast with Study 1, we found an effect of social anxiety on the mimicry of smiles. Participants with a higher level of social anxiety mimicked the social smile of the assistant (AU12, Lip Corner Puller) more, however, the mimicry of enjoyment smiles (or Duchenne smiles; Ekman et al. 1990), including AU6 (Cheek Raiser) was not related to social anxiety. Frowning (AU4) was also not related to social anxiety, consistent with the findings in Study 1.

We could not replicate results from previous studies that socially anxious participants were rejected by others (e.g., Voncken and Dijk 2013; Voncken et al. 2008), because we did not find a relationship between social anxiety level and how likeable participants were judged by their interaction partners. This might indicate that socially anxious individuals are not liked less than healthy controls. Alternatively, the absence of negative judgments of socially anxious participants could be explained by our experimental procedure. Because we did not want to interfere with the muscle movements in the lower face by too much talking, we asked the participants to just listen to the assistant. In other words, the participants received explicit instructions on how to behave and they did not have to do anything special. Because several previous studies have shown that socially anxious participants were especially more negatively evaluated after unstructured tasks in which the social expectations were not so clear (e.g., Thompson and Rapee 2002; Voncken and Dijk 2013), this could also explain the absence of an effect.

## General Discussion

We examined whether individuals’ mimicry and contagion of others’ emotions were related to their level of social anxiety. With regard to emotional mimicry we had two alternative hypotheses, the first stating that social anxious individuals would show overall reduced emotional mimicry, and the second that they would not show reduced, but enhanced mimicry, but only with respect to positive emotions. We conducted two studies in which we used two different paradigms. In Study 1 we examined emotional reactions towards short video clips of standardized emotional faces, whereas Study 2 was designed to examine mimicry in a more interactive setting.

The results of Study 1 showed that participants mimicked the facial expressions of happiness and anger (not fear and contempt), but we did not find any support for the influence of social anxiety. In Study 2, however, we found that social anxiety was related to enhanced mimicry of polite or social smiling (AU12). These latter results are consistent with studies showing that socially anxious individuals tend to display polite smiles (characterized by pulling of the lip corners only) more often than individuals without social anxiety (e.g., Dimberg and Thunberg 2007). These results also complement the findings reported by Heerey and Kring (2007) who showed that socially anxious individuals were less inclined to mimic enjoyment smiles. Our finding of enhanced mimicry of social smiling may be explained by the fact that individuals with social anxiety are more sensitive to socially awkward situations and may show a polite smile in an attempt to appease others, given that they more often fear that they are evaluated negatively (e.g., Clark and Wells 1995). The enhanced display of polite smiles by socially anxious individuals could also be indicative of the perception of their own lower social status. That is, from a biopsychological perspective the enhanced use of polite smiles could be an attempt to avoid conflict with a more dominant other (Deutsch 1990; Gilbert 2000; Ketelaar et al. 2012).

With regard to emotional contagion, the findings were in line with other research on social anxiety and emotions (e.g., Brown et al. 1998; Kashdan and Steger 2006; Moscovitch et al. 2010). Study 1 showed that more socially anxious participants reported more intense irritation in response to all negative facial displays, which is in line with questionnaire and diary studies, showing that at least a substantial sub-sample of socially anxious individuals are inclined to react with anger in daily life (Erwin et al. 2003; Kashdan and Collins 2010; Moscovitch et al. 2008). Social anxiety was also related to more nervousness and irritation, and less happiness, irrespective of the others’ emotion. The enhanced experience of these negative emotions was not reflected in the facial expressions of more anxious individuals which may also suggest that socially anxious individuals suppress their emotion-expression more often, as found in self-report studies (Spokas et al. 2009; Turk et al. 2005).

To date, no other studies have examined the relation between social anxiety and emotional mimicry and contagion with different, standardized paradigms. Both studies show deviating patterns in facial mimicry and/or emotional contagion that seem specific for individuals with social anxiety. The differences that we found are small, and seem to depend on the nature of the social situation enacted in the experiment. This may also partly explain why inconsistent results have been reported: whether individuals with social anxiety mimic, or are caught by others’ emotions, may depend on their relationship with the other person, the task at hand, and the expectation of being evaluated or not. We did not find a relation between social anxiety and emotional mimicry in Study 1, which includes a non-social and non-interactive context. In Study 2, where participants actually interacted, however, we did find a positive relation between social anxiety and the mimicry of polite smiles. This minimally suggests that individuals with higher social anxiety are more likely to respond to an appeasement signal by another person.

The current studies also have some limitations. First, several non-significant (secondary) results could be due to low power. For example, the main effect of social anxiety on the amount of smiling during the computer task in Study 1, would probably have reached significance with an increase in sample size. The same could be true for the negative relation between social anxiety and judgments of likeability in Study 2. Nevertheless, it is unlikely that the main questions in the current studies could not be answered due to power issues. Based on the medium to large effect sizes of previous studies (Dimberg 1997; Khvatskaya and Lenzenweger 2016; Vrana and Gross 2004), a sample size of 32 participants should have been sufficient to test the effect of social anxiety on mimicry. Also, the current effect sizes of the effect of social anxiety on mimicry were very small. The use of computerized coding allowed us to measure facial expression in a non-intrusive manner. However, this method is clearly less sensitive than the use of fEMG and might be especially useful in studies with strong expressions that occur in relative short time frames (as in Study 2). The use of large blocks of facial expressions (as in Study 1), while sitting alone behind a computer, might have resulted in changes in facial muscles that were too subtle and short for the program to register. A second limitation may be the inclusion of students with various levels of social anxiety rather than a clinical population. Although previous studies have shown differences between high and low anxious individuals with similar samples (e.g., Heerey and Kring 2007), it could be that specific deviances in social behavior only occur in individuals with very high levels of social anxiety. These results should therefore be treated with caution before extending to clinical populations.

To summarize, the current studies found that more socially anxious individuals did not show reduced mimicry of negative emotions, but did show enhanced mimicry of polite smiles. At the same time, socially anxious individuals experience more irritation in response to others’ emotions, and more nervousness and less happiness in general. This pattern of findings suggests that socially anxious individuals suppress their negative emotions in social interactions, masking them with a polite smile, potentially to avoid conflict or rejection.

## Footnotes

1 In both studies, we included additional questions about other emotions, attention, and empathy for exploratory purposes. These are not used in the analyses and therefore are not described here. A full account of these measurements can be obtained from the first author.
